# A Mobile Health Intervention to Reduce Sedentary Behavior Among Adolescents and Early Young Adults Receiving Treatment for Acute Lymphoblastic Leukemia: Protocol for a Single-Arm Feasibility Trial

**DOI:** 10.2196/107278

**Published:** 2026-10-28

**Authors:** Brittany J Ivory, Yueh-Yun Chi, Britni R Belcher, David R Freyer

**Affiliations:** 1 Cancer and Blood Disease Institute, Children's Hospital of Los Angeles, 4650 Sunset Blvd, MS #54, Los Angeles, CA, 90027, United States, 1 323-361-3097; 2 Department of Pediatrics, Keck School of Medicine, University of Southern California, Los Angeles, CA, United States; 3 Department of Population and Public Health Sciences, Keck School of Medicine, University of Southern California, Los Angeles, CA, United States; 4 Department of Medicine, Keck School of Medicine, University of Southern California, Los Angeles, CA, United States

**Keywords:** sedentary behavior, sedentary time, physical activity, childhood cancer, adolescent and young adult cancer, acute lymphoblastic leukemia, mobile health, feasibility study, ALL, AYA

## Abstract

**Background:**

Sedentary behavior (SB) is highly prevalent among adolescent and early young adult (eAYA) patients with acute lymphoblastic leukemia (ALL) during treatment and contributes to cardiometabolic complications and morbidity among long-term survivors. SB is distinct from physical inactivity and independently associated with adverse health outcomes, yet few interventions in pediatric oncology have directly targeted reducing SB.

**Objective:**

The RESET in ALL trial was designed as a two-part study to address this research gap. This protocol describes part 1 (1A and 1B, feasibility trial and optimization phase, respectively), including trial design, analysis plan, and anticipated approach to intervention refinement.

**Methods:**

Part 1A was a 12-week, single-arm feasibility trial evaluating a multicomponent mobile health intervention to reduce SB among 20 eAYA patients (ages 12‐21 y) with ALL receiving maintenance therapy at Children’s Hospital Los Angeles. The intervention included a Fitbit tracker with inactivity-triggered prompts, 3 individualized coaching sessions, and a novel app-based peer support group. Primary outcomes were feasibility (retention, enrollment, and tracker wear time) and acceptability (exit surveys and interviews). Secondary outcomes were accelerometer-measured sedentary time and self-reported sitting time. Exploratory outcomes were cardiometabolic biomarkers, anthropometrics, health-related quality of life, and psychosocial and environmental correlates of SB. Part 1B was a synthetic step in which participant feedback and outcome data from part 1A guided optimization of the intervention prior to being tested via a randomized controlled trial in the future part 2 trial.

**Results:**

The study received initial funding in June 2024 and additional funding in July 2025. Participant enrollment and data collection began in September 2024 and were completed in December 2025. Participant enrollment, data collection, and data analysis have been completed. The manuscript reporting the primary trial outcomes is currently under peer review.

**Conclusions:**

This protocol describes the first multicomponent mobile health intervention to reduce SB metrics in eAYAs undergoing cancer treatment. Part 1 (1A and 1B) of the RESET in ALL trial provides the foundation for the development and feasibility evaluation of a novel on-therapy SB intervention, integrating wearable technology, individualized coaching, and peer support. Using a sequential design, in which prior trials inform the optimization of subsequent trials, we aim to develop and refine a scalable, clinically integrated approach to behavior change during treatment that enhances health outcomes in eAYA populations.

## Introduction

Acute lymphoblastic leukemia (ALL) is the most common malignancy in children, adolescents, and early young adults (eAYAs, age 15‐21 y). Due to recent advances in treatment, 5-year survival for this group now exceeds 90%, leading to a growing population of long-term survivors [1]. ALL survivors are at increased risk for obesity, impaired glucose metabolism, diabetes mellitus, low bone mineral density, reduced physical fitness, and cardiovascular disease, which contribute to long-term morbidity and early mortality [2-9]. These risks are compounded by high levels of physical inactivity and sedentary behavior (SB), which are associated with adverse cardiometabolic outcomes [10-12].

SB is defined as any low-intensity (≤1.5 metabolic equivalents of task [METs]) waking activity performed in a seated, reclined, or lying position and is distinct from physical inactivity, defined as insufficient moderate-to-vigorous physical activity (MVPA) [13,14]. Notably, SB and MVPA are only weakly correlated, meaning an individual can meet MVPA guidelines while still accumulating high levels of sedentary time [13,14]. SB becomes highly prevalent among childhood and eAYA patients with cancer early during treatment, with an estimated 80% of waking hours spent sedentary [15-17]. ALL therapy is commonly complicated by deconditioning and cardiometabolic effects, which can be exacerbated by excessive SB and lead to poorer outcomes (eg, lower survival and increased risk for relapse) [6,18-20]. In addition, studies of childhood and eAYA patients with cancer have shown that higher levels of SB during treatment are associated with lower cardiorespiratory fitness and poorer quality of life (QoL) [15,21]. These lifestyle behaviors often persist following therapy, leading to long-term sedentarism and associated risk of morbidity and mortality [2,15,22]. Therefore, intervention strategies to mitigate the short- and long-term negative health effects of SB during treatment are needed for this high-risk population.

Reducing SB with brief light- or moderate-intensity physical activity (PA) leads to improved short-term metabolic function and QoL (eg, reduced negative affect) in healthy children [23,24]. Recent systematic reviews indicate that replacing sedentary time with just 30 minutes per day of light-intensity PA can lead to improvement in cardiometabolic biomarkers and reduced adiposity [25-27]. Thus, these early studies provide preliminary support that reducing SB may also elicit beneficial effects in youth with cancer.

Despite this, there remain significant literature gaps in interventions that specifically target reductions in SB in children and eAYAs with cancer. First, most interventions in pediatric oncology have focused on increasing PA in long-term survivors [28]. However, intervention strategies to increase PA do not necessarily lead to reduced SB, and it is recommended that SB be specifically targeted using separate intervention strategies [29]; this is supported by evidence showing most PA interventions in childhood cancer survivors (CCS) have not shown a significant reduction in SB [12]. Second, most interventions begin after treatment has concluded, by which time unhealthy lifestyles are often already ingrained. The maintenance phase of ALL therapy presents an optimal window for intervention, as acute toxicities begin to subside and patients regain capacity for regular activity [30]. Benefits of intervening during this phase may include greater likelihood of sustained behavior change and enhanced self-efficacy as patients transition to survivorship. Especially for patients still on therapy, SB interventions are likely to be more feasible and acceptable than PA interventions due to the abundant opportunities for SB replacement throughout the day and greater appeal of short bouts of light-to-moderate activity not requiring special equipment, training, or skills.

Finally, few interventions have targeted eAYAs, a high-priority population for behavior change interventions because lifestyle habits established during this developmental period often track into adulthood [31,32]. Smartphone use is widespread among eAYAs, making mobile health (mHealth) interventions an appealing strategy for promoting behavior change. These tools can reduce common barriers to participation (eg, logistics of travel) and enable real-time data collection and feedback, especially when paired with wearable activity trackers [33,34]. mHealth approaches have effectively reduced SB in healthy youth [35,36] and have been piloted in CCS [37,38].

To address these research gaps and inform future studies, we are conducting a two-part clinical trial, “RESET in ALL,” testing a novel mHealth SB intervention in eAYAs with ALL during maintenance therapy. Part 1 included: (1A) a 12-week, single-arm trial to establish initial feasibility and acceptability of a multicomponent SB intervention, including a wearable fitness tracker with inactivity-triggered prompts, an app-based peer support group, and individualized coaching sessions; and (1B) intervention optimization based on feasibility trial results, including direct participant feedback. In the future part 2, the optimized SB intervention will be tested in a pilot randomized control trial (RCT) to assess preliminary efficacy and effect size. Results from this innovative two-part design will yield a data-driven, participant-informed intervention, which can be fully tested for efficacy in a future definitive trial (Figure 1). This paper describes the rationale, study design, statistical analyses, and intervention optimization plan for part 1 (A and B) of the RESET in ALL trial.

**Figure 1.**
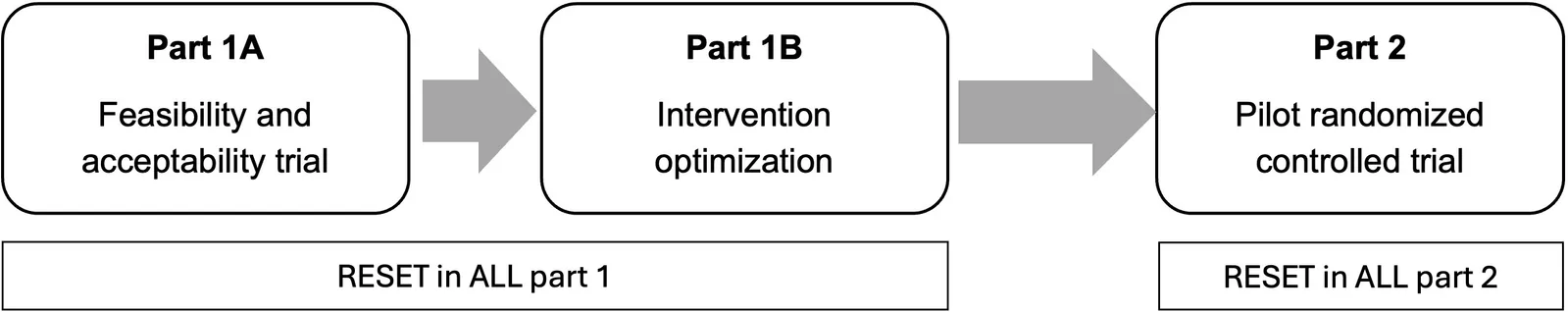
Conceptual model for two-part intervention in eAYAs with ALL during maintenance therapy, the “RESET in ALL” study. ALL: acute lymphoblastic leukemia.

## Methods

### Study Design

RESET in ALL part 1A was a single-arm, 12-week feasibility trial in which all participants received the intervention to optimize evaluation of intervention feasibility and acceptability. Target enrollment was 20 participants. The trial is registered at ClinicalTrials.gov (NCT06182163), and the registration record includes all items from the World Health Organization Trial Registration Data Set. The study schema for the part 1A trial is shown in Figure 2.

**Figure 2.**
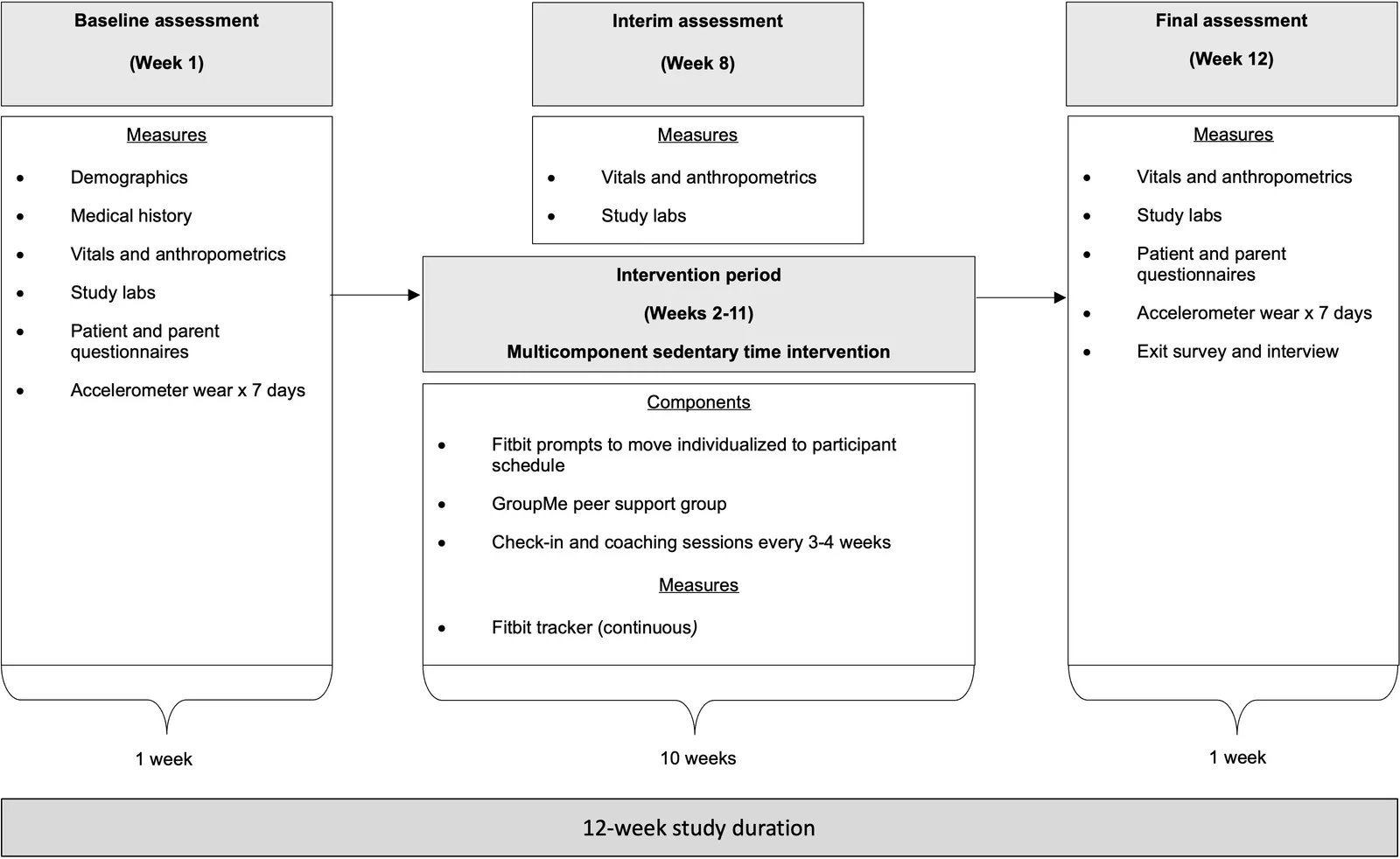
Study schema for RESET in ALL part 1A.

### Specific Aims

The primary aims of the RESET in ALL part 1A trial were to establish the feasibility and acceptability of a multicomponent mHealth intervention among eAYA (12‐21 y) patients with ALL receiving maintenance therapy at Children’s Hospital Los Angeles (CHLA). A secondary aim was to evaluate the effects of the intervention on objectively measured SB, assessed via accelerometer-measured sedentary time and time accrued in uninterrupted sedentary bouts ≥1 hour (min/d), as well as self-reported sitting time (min/d). Exploratory aims were to assess intervention effects on the following: (1) clinical and lab biomarkers of cardiometabolic health; (2) health-related quality of life (HRQoL); and (3) associations between psychosocial and environmental factors and change in SB metrics from preintervention to postintervention.

### Eligibility and Recruitment

This study was conducted at CHLA in Los Angeles, CA, which serves a large, sociodemographically diverse patient population. Based on institutional experience, 1 to 3 eAYA patients with ALL per month were expected to meet eligibility criteria and enroll in the study. We expected our study population to be approximately 65% White Hispanic, 17% White non-Hispanic, 4% Asian, 5% Black, and 9% Pacific Islander, Native American, or Other. In addition, about two-thirds of CHLA oncology patients fall within the lowest 40% of socioeconomic status, and approximately 70% are covered by Medicaid (Medi-Cal/California Children’s Services).

Eligibility criteria are listed in Textbox 1. The age range of 12‐21 years was chosen as this group was developmentally appropriate to engage with the intervention and to participate together in an online peer support group based on shared life experiences (school, early jobs, etc). Patients with B-cell lymphoblastic lymphoma (B-LLy) or T-cell lymphoblastic lymphoma (T-LLy) and mixed phenotype acute leukemia (MPAL) were also eligible due to treatment similarities with ALL [39,40]. Patients were enrolled after completing at least 1 month of maintenance therapy (cycle 1 day 29) to allow for resolution of potential adverse effects from upfront intensive therapy; in addition, participants were required to have 1 cycle (3 mo) of therapy remaining to ensure that all participants were receiving active therapy at the time of study. Participants were eligible if they self-reported at least 4 hours per day of leisure-time SB, given this is the lower threshold of time spent in SB reported in studies among healthy children and adolescents [31,41].

Due to limited resources, the intervention could only be offered in English; thus, participants needed to be able to speak and read English, which aligns with the language fluency of nearly all CHLA patients from school age onward. Parents or guardians of participants under 18 years of age did not need to be English-speaking, as consent and surveys can be performed in the parent’s preferred language with the assistance of a certified medical interpreter. Exclusion criteria were related to medical or cognitive conditions that would affect a participant’s ability to fully engage in the intervention.

Participants were recruited through the oncology clinics of CHLA’s Cancer and Blood Disease Institute (CBDI). Clinic schedules were screened by the study principal investigator (PI; Ivory), and providers for potentially eligible participants were contacted for approval to approach the patient in the clinic. If approved, study staff approached the patient during their next clinic visit to assess interest and confirm eligibility. IRB-approved recruitment flyers were also disseminated in the oncology clinics for patient or parent viewing, and oncology providers were informed of trial eligibility criteria and objectives to promote patient referrals.

Textbox 1. **Eligibility criteria.**

**Inclusion criteria**

- 12-21 years of age at time of enrollment
- Diagnosis of leukemia/lymphoblastic lymphoma treated with COG-based maintenance chemotherapy (B-cell acute lymphoblastic leukemia/lymphoma [B-ALL/Ly], T-cell acute lymphoblastic leukemia/lymphoma [T-ALL/Ly], and mixed phenotype acute leukemia [MPAL])
- Has completed at least one month of maintenance chemotherapy (through maintenance cycle 1, day 29) and has at least one cycle (3 mo) remaining
  - Of note: patients treated with nelarabine are eligible to enroll during nelarabine-containing cycles of maintenance therapy.
- Baseline sedentary lifestyle with a self-reported average of 4 or more leisure-time hours per day spent sedentary (verbal report)
- Has access to the internet and smartphone compatible with Fitbit and GroupMe (own or parent’s or legal guardian’s)
- Fluent in English (participant only)

**Exclusion criteria**

- Evidence of recurrent or metastatic disease
- Medical contraindication to daily standing and light physical activity (>1.5 metabolic equivalents of task [METs])
- Intellectual disability or developmental delay that limits ability to fully participate in the study intervention

### Ethical Considerations

This protocol was developed in accordance with the SPIRIT (Standard Protocol Items: Recommendations for Interventional Trials) 2022 guidelines (Checklist 1) [42], and study results will be reported according to the CONSORT (Consolidated Standard of Reporting Trials) 2010 extension for pilot and feasibility trials [43]. All study procedures were reviewed and approved by the CHLA Institutional Review Board (IRB No. CHLA-23‐00346), and annual continuing reviews are ongoing during the data analysis phase. All protocol modifications were submitted for IRB review, and when relevant, study changes were communicated to participants in-person at study visits or via phone. Protocol versions 1‐6 were comprised of editorial changes during the initial IRB submission. Protocol versions 7‐11 comprised the following amendments to the approved protocol: (version 7) changed eligibility criteria to include participants with diabetes and/or autoimmune or rheumatologic disorders, defined which leukemia diagnoses were eligible for the study, and added clarification on timing of eligibility for patients treated with nelarabine; (version 8) changed the app platform for the peer support group to enhance participant privacy; (version 9) increased the upper age limit from 18 to 21 years to increase the number of eligible participants, changed consent procedures to include over the phone or Microsoft Teams, and added a parent exit interview survey; (version 10) reduced total number of participants from 30 to 24 to meet our study timeline requirements and changed eligibility criteria to allow participants to enroll in the study earlier on during treatment; and (version 11) updated the funding and study PI surname. Multimedia Appendix 1 provides details on changes between protocol versions and corresponding updates to the study record on ClinicalTrials.gov.

Informed consent was obtained from all individual participants ≥18 years old or from a parent or legal guardian for adolescents <18 years old by the study PI. Assent was also obtained from all participants less than 18 years old. In the case of parents or guardians who were not English-speaking, an approved short form in their preferred language was provided with the English consent form, and the informed consent process was conducted with a certified medical interpreter. Health Insurance Portability and Accountability Act (HIPAA) authorization was obtained from the parent or legal guardian (in their preferred language) or adult participant at the time of informed consent, given the plan for abstraction of oncologic history from the electronic medical record (EMR). Participants received a US $50 Amazon gift card as compensation for completing the study.

Given that this was a single-center feasibility trial with minimal anticipated risks, a formal Data Safety Monitoring Board was not established. Oversight and auditing functions were performed by the PI, including regular review of study data, evaluation of protocol adherence, and safety assessments as needed. No study-related adverse events occurred.

### Setting and Timeline

#### Study Procedures

Study participation from baseline to study completion was about 12 weeks in duration (over 1 week for final accelerometer assessment, if needed), which aligns with one 12-week cycle of maintenance therapy per Children’s Oncology Group (COG)-type protocols. All participants completed 3 in-person study visits in the hematology and oncology clinics at CHLA at baseline, week 8, and week 12 (Figure 2). To reduce participant burden, these were scheduled to coincide with routine monthly maintenance chemotherapy visits; blood draws were performed at the CHLA outpatient lab in conjunction with clinical testing.

During the baseline visit, written informed consent and assent were obtained, if not completed previously, and participants received baseline materials, including study questionnaires and the thigh-mounted ActivPAL accelerometer, initialized and applied by study staff. Participants were also issued a wrist-worn Fitbit device, which was not activated until the orientation visit. Following completion of accelerometer wear (week 2), a virtual orientation session was held via a secure group-meeting platform (eg, Microsoft Teams). During this session, participants were given a Fitbit account using anonymized credentials, oriented to use of the Fitbit app and tracker, and added to the app-based peer support chat. The first individualized coaching session was completed during the orientation. Additional coaching occurred virtually at week 4 and in person at week 8. At the week 12 visit, accelerometers were collected or initialized and applied, as applicable (depending on week 11 vs 12 wear period); postintervention questionnaires were completed, and participants were invited to take part in an optional exit interview.

#### Intervention

The study intervention was based on principles of the Health Action Process Approach (HAPA) model of behavior change, which has been shown to be effective in modifying SB in adolescents and university students [44,45]. It posits that there is a motivation phase, where an intention to change behavior is made, as well as a volition phase in which actions are taken to change and maintain behavior [46]. Given that our intent was to support action to reduce SB, we focused on key constructs of the volition phase to build our intervention, including action planning, coping planning, and action control, which were significantly associated with reduction in SB in university students [44], as well as self-efficacy. Participants were encouraged to reduce their overall SB by 30 minutes per day through achievable, light-intensity activities throughout the day. The intervention included three synergistic mHealth components to support participants in meeting this goal.

#### Wearable Fitness Tracker With Prompts to Move

Participants received a Fitbit Charge 6 tracker (Google LLC) programmed with hourly, inactivity-triggered haptic prompts, which are included with the Fitbit app (Google LLC). “Reminder to move” prompts were delivered if fewer than 250 steps were taken in the preceding 50 minutes, accompanied by reinforcement messages if movement goals were achieved. Each participant selected a daily 10-hour prompting schedule, individualized to accommodate daily routines. For participants attending school, a 6-hour window during afternoon-evening leisure time was chosen for participants to focus on responding to the prompts. Participants set goals for hourly activity (ie, number of hours out of 10 that they will take at least 250 steps) based on their baseline SB level, with support from study staff during the orientation visit. Participants were able to view real-time feedback on their activity levels on the Fitbit tracker and app to help them monitor their progress and achievements. Participants were allowed to keep their Fitbit after completing the study. Several studies have safely used Fitbit trackers for the collection of activity data and/or PA promotion in childhood and AYA patients with cancer and survivors [47].

#### Personalized Coaching Sessions

Each participant completed 3 individualized coaching sessions with trained study staff: a virtual session during the intervention orientation visit (week 2), a virtual session at week 4, and an in-person session at week 8. Sessions incorporated a review of Fitbit data to support goal-setting, action planning, and coping strategies, based on behavior change principles from HAPA [46]. Sessions were tailored to participants’ individual barriers, preferences, and family support systems. Parents or guardians were encouraged but not required to attend sessions.

#### App-Based Peer Support Group

Participants were enrolled in a private, study-specific GroupMe chat (Microsoft Corporation, Redmond, WA) using anonymized usernames. Weekly discussion prompts were posted by study staff to encourage engagement, normalize challenges, and foster peer support related to reducing SB. Participants were encouraged to share experiences, strategies, and encouragement with each other. The group was monitored by study staff to ensure safety and appropriateness of content, and engagement was tracked through app usage data and participant self-report. Use of an app-based peer support group was a novel approach tailored for the developmental stage of adolescence, where peer relationships are critical for motivation and normalization of behavior [48]. This approach was chosen over social media platforms, such as Instagram, Facebook, and TikTok, in order to limit additional screen time, which is highly associated with SB [49], and based on research indicating that excess social media usage is associated with decreased self-esteem and poor mental health [50]. Interest in different social media platforms also tends to wane over time, which may decrease the sustainability of interventions based on such programs.

### Concomitant Care and Interventions

Participants continued to receive all usual medical and supportive care during the study, including physical and occupational therapy. However, they were asked not to enroll in other structured PA or SB interventions during the study period to minimize contamination of secondary and exploratory outcomes.

### Participant Withdrawal

Participants were able to withdraw from the study at any time upon request or at the discretion of the study PI or treating oncologist if continued participation was deemed inappropriate (eg, due to disease relapse, change in medical status, hospitalization, or treatment complications). Participants who discontinued the intervention prematurely were invited to complete a study exit visit, including final outcome assessments, whenever possible. Participants received gift card compensation scaled to their duration of participation (ie, US $15 for ≤1 mo or US $30 for 1‐3 mo).

### Measures

#### Primary Outcomes

##### Feasibility

Table 1 provides an overview of study outcomes and measurement timings. The primary end points of the RESET in ALL part 1A trial were intervention feasibility and acceptability. Feasibility was primarily defined by a study retention rate of ≥70%, based on completion of study questionnaires at both baseline and week 12 assessments. Additional predefined feasibility benchmarks are as follows: (1) enrollment of up to 24 eligible participants in order to ensure there will be 20 evaluable participants, defined as those who completed a preintervention and postintervention SB assessment (accelerometer and/or self-report) and a postintervention exit survey; and (2) ≥50% of participants wearing the Fitbit tracker for at least 10 waking hours on ≥70% of intervention days. Retention was the primary criterion for feasibility, with enrollment and device adherence evaluated as secondary indicators to inform feasibility assessment and guide future trial design.

**Table 1.** RESET in ALL part 1 study outcome assessments, timing of assessments, and role in intervention optimization.

Study outcomes and measurement	Description	Timing of assessment	Role in intervention optimization	Reference
Primary outcomes				
Feasibility				
Retention proportion	Percentage of participants who complete baseline and week 12 assessments; goal: ≥70% (primary)	Study closure	Determine if modifications are needed to optimize participant retention and engagement.	—^a^
Study enrollment	Number of eligible patients who enroll in the study; goal: 20 evaluable	Study closure	Provide information on patient interest in the intervention and ability to enroll efficiently, and inform efforts to enhance recruitment.	—
Fitbit tracker wear time	Percentage of participants who wear a Fitbit tracker for at least 10 waking hours on ≥70% intervention days; goal: ≥50%	Continuously from week 2 through week 12	Determine if targeted intervention is needed to increase Fitbit wear time.	—
Acceptability				
Exit survey	12-item study-specific tool with Likert scale and short answer prompts on overall intervention and individual components (primary)	Week 12	Inform changes to the overall three-part intervention and to each individual component, as applicable, to enhance engagement and effectiveness.	—
Exit interview (optional)	Semistructured, recorded 10-minute to 15-minute interview for detailed participant feedback	Week 12	Detailed and directed feedback to inform changes to the overall three-part intervention and each individual component, as applicable, to enhance engagement and effectiveness.	—
Engagement in group chat	Approximate number of posts read, post reactions, and new posts per participant	Week 12	Provide summary information on engagement in group chat, determine if group chat should continue to be used, and if yes, changes needed to enhance participation.	—
Secondary outcomes				
Sedentary behavior				
Device-measured				
ActivPAL micro4	Thigh-mounted accelerometer, which measures 7 days of 24-hour movement behaviors (eg, SB^b^, PA^c^, etc)	Weeks 1 and 12	Provide early data on the effect of the intervention on SB and if changes should be made to SB reduction approach.	[51,52]
Fitbit Charge 6	Wrist-worn fitness tracker with continuous measurement of SB and PA via steps and heart rate	Continuously from week 2 through week 12	Supplemental data on longitudinal change in SB to inform changes to SB reduction approach, if indicated.	[50]
Self-report				
International Physical Activity Questionnaire (IPAQ)	Validated self-report tool to measure weekday and weekend sitting time	Baseline and week 12	Provide early data on effect of intervention on SB and if changes should be made to SB reduction approach.	[53]
Exploratory outcomes				
Cardiometabolic biomarkers				
Glucose metabolism				
Hemoglobin A_1c_	Blood test that reflects average glucose over 2‐3 months	Baseline and week 12	Provide early data on effect of intervention on glucose metabolism and usefulness of this measure for future study.	[54]
Fructosamine	Blood test that reflects average blood glucose over 2‐4 weeks	Baseline, week 8, and week 12	Provide early data on effect of intervention on glucose metabolism and usefulness of this measure for future study.	[54]
Systemic inflammation				
High-sensitivity C-reactive protein	Blood test of systemic inflammation that has been associated with SB and cardiovascular risk factors	Baseline, week 8, and week 12	Provide early data on effect of intervention on inflammation and usefulness of this measure for future study.	[55,56]
Lipid metabolism				
Fasting lipid panel (optional)	Blood test to measure levels of cholesterol and triglycerides	Baseline and week 12	Provide early data on effect of intervention on lipid metabolism and usefulness of this measure for future study.	[57]
Vital signs				
Heart rate, blood pressure	Vital signs recorded by medical assistant for clinical oncology visit	Baseline, week 8, and week 12	Provide early data on effect of intervention on objective measures of cardiovascular health.	—
Anthropometrics				
Weight, height, BMI, waist circumference, and waist-to-height ratio (WtHR)	Weight and height measured by medical assistant for clinical oncology visit; waist circumference measured by study staff; BMI and WtHR calculated in REDCap	Baseline, week 8, and week 12	Provide early data on effect of intervention on adiposity and need for incorporation of other measures of body composition.	[58]
Health-related quality of life (HRQoL)				
PedsQL^d^ 4.0 Generic Core Scales	Validated 23-item scale that assesses physical, emotional, social, and school functioning	Baseline and week 12	Provide early data on effect of intervention on HRQoL and if specific intervention measures can be incorporated to promote improvement in HRQoL domains.	[59,60]
PedsQL 3.0 Cancer Module	Validated 27-item scale that assesses physical, mental, and emotional adverse effects from cancer and its treatment	Baseline and week 12	Provide early data on effect of intervention on HRQoL and if specific intervention measures can be incorporated to promote improvement in HRQoL domains.	[59]
PedsQL 3.0 Multidimensional Fatigue Scale	Validated 12-item scale that assesses general fatigue, sleep or rest fatigue, and cognitive fatigue	Baseline and week 12	Provide early data on effect of intervention on HRQoL and if specific intervention measures can be incorporated to promote improvement in HRQoL domains.	[59]
Psychosocial and environmental correlates of sedentary behavior				
PACE^e^ Adolescent Psychological and Sedentary Behavior Survey	Validated survey assessing adolescent attitudes, self-efficacy, decisional balance, and social support related to SB	Baseline and week 12	Evaluate psychosocial correlates of SB that may require targeted intervention and measurement.	[61,62]
PACE Health and Environment Survey for Parents	Validated survey assessing parental support for SB reduction and home and environmental factors that affect SB	Baseline and week 12	Evaluate environmental and social correlates of SB that may require targeted intervention or individualized approaches for SB reduction.	[61]
Other variables				
Demographics				
Self-report questionnaire	Child age, sex, race, ethnicity, type of insurance, household structure (eg, single vs dual parent), maternal education level, zip code	Baseline	Inform intervention modifications that may be needed to accommodate participants’ cultural and/or socioeconomic background.	—
Medical history				
Medical record abstraction	Leukemia type, date of diagnosis, relevant treatment history and adverse effects, chronic disease, days of hospitalization	Baseline	Inform specific intervention approaches that may be needed based on participant treatment history and adverse effects.	—

a Not applicable

b SB: sedentary behavior.

c PA: physical activity.

d PedsQL: pediatric quality of life inventory.

e PACE: patient-centered assessment and counseling for exercise.

##### Acceptability and Participant Feedback

Acceptability and perceived effectiveness were assessed with an internally developed, study-specific exit survey (Table 2) that was completed by all participants and optional semistructured exit interviews. The survey included Likert-scale and open-ended questions evaluating the program overall and individual components (ie, Fitbit, coaching, and group chat). Exit interviews were conducted to gather more in-depth participant feedback on the program and suggestions for improvement, with a target of 10 interviews (out of 20 participants). Interviews were audio-recorded, transcribed by a third-party service, and deidentified using study IDs. Parent surveys and interviews were optional and used to inform future studies but did not factor into primary acceptability outcomes. For parents who did not speak English, surveys and interviews were conducted verbally with a certified interpreter in their preferred language.

**Table 2.** RESET in ALL part 1 participant exit survey for intervention acceptability and perceived effectiveness.

Item number	Survey prompt	Response options
1	It is important for my health to reduce my daily sedentary time (time spent sitting or lying down and inactive, such as watching TV, using the computer, or playing video games).	Strongly disagree, disagree, agree, and strongly agree
2	Participation in this study helped me to reduce my daily sedentary time.	Strongly disagree, disagree, agree, and strongly agree
3	The Fitbit and reminders to move helped me to reduce my daily sedentary time.	Strongly disagree, disagree, agree, and strongly agree
4	The coaching sessions with study staff helped me to reduce my daily sedentary time.	Strongly disagree, disagree, agree, and strongly agree
5	The GroupMe chat with other study participants helped me to reduce my daily sedentary time.	Strongly disagree, disagree, agree, and strongly agree
6	I plan to continue to work on reducing my daily sedentary time over the next 3 months.	Strongly disagree, disagree, agree, and strongly agree
7	I would recommend this program to other teenagers and young adults being treated for cancer.	Strongly disagree, disagree, agree, and strongly agree
8	What was the most helpful part of the study program?	Free-text response
9	What was the least helpful part of the study program?	Free-text response
10	What was your favorite part of the study program?	Free-text response
11	What was your least favorite part of the study program?	Free-text response
12	Did you make any changes to your diet (what you eat) during the study?	Yes, no
13	If yes, what changes did you make in your diet?	Free-text response

### Secondary Outcome: Sedentary Behavior

SB was objectively measured at baseline and postintervention using the ActivPAL micro4 triaxial accelerometer (PAL Technologies Ltd). The device yields valid estimates of sedentary time, postural changes, and light-to-moderate PA in 60-s epochs and has been validated for SB assessment in adolescents [63,64]. Participants wore the device mounted on the right thigh continuously for 7 days directly following the baseline visit and during weeks 11 or 12 (before or directly after the final study visit). At both time points, data were downloaded and screened for completeness using the PAL Software Suite (PAL Technologies Ltd). When possible, participants were asked to rewear the accelerometer if it was not worn for ≥4 days with ≥10 waking hours per day. Data was processed using the PAL Software Suite; measures of primary interest for this study included total sedentary time (min/d), sitting time (min/d), time accrued in uninterrupted sedentary periods >1 hour (min/d), and steps per day. To complement device-based measures, participants also self-reported daily sitting time using the validated International Physical Activity Questionnaire (IPAQ) long-form [51].

Throughout the 10-week intervention, SB and PA were assessed via the Fitbit tracker and monitored using the Fitabase analytics system (Small Steps Labs LLC), a secure online platform that aggregates Fitbit data for research use. Fitabase was used to synthesize and manage data on tracker wear time, heart rate, steps/day, and hours/day with <250 steps. Fitbit wear time was determined by the presence of heart rate data, which was measured continuously by the tracker and recorded in Fitabase.

### Exploratory Outcomes

#### Cardiometabolic Biomarkers

Weight, height, waist circumference, resting heart rate, and blood pressure were recorded at baseline, week 8, and week 12 assessments. Vital signs, height, and weight were measured once by a licensed medical assistant as per the standard of care for oncology clinic visits. A stadiometer was used to measure the participant’s height to the nearest 0.1 cm. Weight was measured on a digital scale to the nearest 0.1 kg. Waist circumference was measured using a flexible, nonstretchable measuring tape at the midpoint between the lowest palpable rib and the top of the iliac crest. The measurement was recorded to the nearest 0.1 cm. Body mass index (kg/m2; BMI) and waist-to-height ratio (WHtR) were calculated to assess adiposity [52].

Laboratory biomarkers included fructosamine (baseline, week 8, and week 12) and hemoglobin A_1c_ (HbA_1c_; baseline and week 12) to evaluate trends in glucose metabolism. Fructosamine is a short-term measure of glucose control that reflects average blood glucose levels over the prior 2-week to 4-week period and correlates with fasting and postprandial glucose. It has been shown to be useful when monitoring glucose control during short-term exercise [53]. Neither HbA_1c_ nor fructosamine require fasting for accurate measurement [53]. High-sensitivity C-reactive protein (hs-CRP) was measured given its association with cardiovascular risk [58] as well as sedentary time in children [54]. An optional fasting lipid panel was collected as an additional marker of cardiometabolic health at baseline and week 12 assessments when feasible [55]. We aimed to obtain fasting lipid measurements from at least half of participants.

#### Patient-Reported Outcomes

HRQoL was measured at baseline and week 12 using the self- and parent-report versions of the validated and widely used Pediatric Quality of Life Inventory (PedsQL) 4.0 Generic Core Scales, 3.0 Cancer Module, and 3.0 Multidimensional Fatigue Scale [56,57]. The Generic Core Scales consist of 23 items across four domains: (1) physical functioning, (2) emotional functioning, (3) social functioning, and (4) school functioning. The Cancer Module includes 27 items covering eight domains: (1) pain and hurt, (2) nausea, (3) procedural anxiety, (4) treatment anxiety, (5) worry, (6) cognitive problems, (7) perceived physical appearance, and (8) communication [56]. Finally, the Multidimensional Fatigue Scale contains 18 items across three domains: (1) general fatigue, (2) sleep or rest fatigue, and (3) cognitive fatigue. For all 3 scales, higher scores indicate better HRQoL [56]. Age-specific versions were used as designed. Parent participation was optional for adults ≥18 years. Spanish versions were provided for parents when applicable.

#### Psychosocial and Environmental Correlates of SB

For exploratory evaluation of factors influencing SB, participants completed the PACE Adolescent Psychological and SB Survey at baseline and week 12 assessments [59,60]. Parents or guardians (or adult participants) completed the PACE Health and Environment Survey for Parents [59]; for those who were not English-speaking, the survey was completed verbally with the assistance of a certified interpreter. These validated tools measure self-efficacy, behavior change strategies, social influences, and neighborhood characteristics [59,60].

### Other Variables: Participant Characteristics

Participants and their parents or guardians self-reported demographic information, including race, ethnicity, zip code of primary residence, number of parents in the household, and maternal education level. Clinical and treatment characteristics were abstracted from the electronic medical record and entered into the study database by the study PI and a research assistant. Extracted variables included age at enrollment, sex assigned at birth, leukemia type, date and age at diagnosis, type and estimated cumulative dose of steroids (mg/m²), cumulative vincristine dose (mg/m²), relevant therapy-related adverse effects (eg, steroid-induced hyperglycemia, myopathy, and neuropathy), chronic health conditions (eg, diabetes mellitus, dyslipidemia, and chronic heart disease), and total days of hospitalization.

### Blood Specimen Storage for Future Research

As an optional procedure, residual blood samples from clinical and study laboratory draws at each study visit (ie, baseline, week 8, and week 12) were stored for future research examining biomarkers (eg, cardiometabolic, cancer-specific, etc) associated with SB in patients with ALL. Participation in this procedure was opt-in and required separate consent or assent as part of the initial informed consent process. Specimens were labeled with the participant’s study ID and stored in a secure, access-controlled laboratory freezer. All specimens were managed directly by the study PI in accordance with institutional biorepository policies.

### Statistical Analyses

#### Data Collection and Integration

Study data was collected and managed using REDCap electronic data capture tools hosted at the University of Southern California; REDCap (Vanderbilt University) is a secure, web-based software platform designed to support data capture for research studies [61,62]. Data was labeled with a unique study ID for each participant, and the key linking study IDs with participant names was kept in a secure, password-protected database available only to the study PI. Questionnaire data was directly entered into REDCap by the participant and parent or transcribed verbatim by study staff if paper surveys were specifically requested or the surveys were conducted verbally with an interpreter. Other data, such as vital signs, anthropometrics, and lab results, were entered into REDCap by study staff. All study data, including accelerometer and Fitbit measures and REDCap records, were analyzed using R (Version 4.5.2; R Foundation for Statistical Computing, Vienna, Austria). Thematic analysis of study exit interviews was performed using Dedoose (SocioCultural Research Consultants LLC).

#### Sample Size Determination

As this was a feasibility trial, formal power calculations were not conducted. The sample size was chosen to provide sufficient data to assess feasibility and acceptability outcomes and to inform power calculations and design of a larger trial.

#### Primary Analysis

Feasibility and acceptability metrics were summarized descriptively. To determine attainment of feasibility criteria, we evaluated: (1) the number of participants who completed study questionnaires at baseline and week 12 out of the total number of participants enrolled (retention); (2) the number of eligible eAYAs who enrolled in the study (enrollment); and (3) the number of days of participant Fitbit wear more than 10 hours out of the total number of intervention days (Fitbit wear time). To determine acceptability, the percent of participants reporting “agree” or “strongly agree” to the exit survey prompt “Participation in this study helped me reduce my daily sedentary time” was evaluated. Acceptability of each individual intervention component was measured in a similar manner as supplemental measures. Engagement in the peer support group chat was summarized using descriptive statistics as a secondary acceptability measure. Finally, major themes from participant and parent exit interviews were summarized through qualitative thematic analysis.

Changes in SB (secondary end point), cardiometabolic biomarkers, and HRQoL (exploratory end points) were summarized with descriptive statistics and evaluated using the Wilcoxon signed-rank test. Median changes with interquartile range (IQR) were described for SB measures between baseline and week 12. Individual participant changes in SB metrics were summarized using waterfall plots. For cardiometabolic outcomes assessed at 3 time points, changes from baseline to week 8 and baseline to week 12 were described using medians and IQRs. The significance threshold was adjusted to account for multiple comparisons. Patterns and extent of missing data, as applicable, were summarized to understand potential reasons for missingness and to guide planning for a larger trial. The associations of psychosocial and environmental factors with changes in SB have not yet been analyzed and will be examined in a future secondary analysis using linear regression models, with log transformation of variables as needed to approximate Gaussian assumptions.

### Intervention Optimization

After completion of primary analysis, results from the part 1A trial were used directly in part 1B, optimization of the RESET in ALL intervention (Figure 1) to enhance efficacy in subsequent trials. Table 1 provides a summary of how part 1A trial outcomes informed intervention optimization in part 1B. Specifically, acceptability of the intervention overall and each individual intervention component (ie, the Fitbit tracker with prompts to move, coaching sessions, and app-based peer support group) determined if modifications were needed to optimize participant satisfaction and engagement. Themes from the study exit surveys and interviews specifically were emphasized. The acceptability of the overall intervention was evaluated for the usefulness of integrating three components, mHealth monitoring and communication, and flexible, individually determined activities to interrupt SBs. Additionally, the need for more integration of parents or a designated support person into the intervention was assessed.

Participant feedback informed refinement of the wearable technology, health coaching, and peer support components, as well as the addition of new intervention features to improve feasibility, relevance, and participant engagement. A board-certified pediatric psychologist with expertise in eAYA oncology guided intervention refinement to enhance participant engagement, psychosocial acceptability, and cultural relevance, as appropriate. The resulting optimized intervention, incorporating these participant-informed refinements and evidence from the published literature, will be described in detail in the protocol for the subsequent RESET in ALL part 2 randomized pilot trial. Finally, psychosocial and environmental survey data, together with demographic and medical history data, will be integrated in a future secondary analysis to identify contextual and individual-level factors that may inform targeted and individualized approaches to reducing SB in this patient population.

### Progression to Part 2

Feasibility and acceptability served as the primary determinants of the appropriateness of progression to the part 2 RCT. We determined that the intervention would be deemed definitively feasible if a retention rate of ≥70% was met and definitively acceptable if ≥60% of participants reported on the study exit survey that participation in the study helped them reduce their sedentary time. In addition, a reduction of approximately 30 minutes/day in sedentary time is considered clinically meaningful based on prior evidence and will serve as a secondary benchmark for evaluating intervention promise and informing progression to part 2. Decisions regarding progression will also consider intervention adherence and findings from secondary and exploratory outcomes.

## Results

This study received initial funding in June 2024 and additional funding in July 2025. Participant enrollment and data collection began in September 2024 and were completed in December 2025. Primary analyses have been completed, with additional secondary analyses ongoing. The manuscript reporting the primary study results is currently under peer review.

## Discussion

### Principal Findings

This protocol describes a multicomponent mHealth trial designed to evaluate preliminary feasibility and acceptability and optimize an intervention to reduce SB in eAYA patients undergoing cancer treatment. We hypothesized that participants would find the intervention feasible and acceptable and would show a signal toward reducing SB metrics over 12 weeks. The sequential study design allows for iterative refinements: part 1A was a 12-week, single-arm feasibility study evaluating a multicomponent mHealth intervention that combined a Fitbit tracker with inactivity-triggered prompts, individualized coaching, and an app-based peer support group. Data from the part 1A trial directly informed intervention optimization in part 1B, particularly acceptability measures and participant feedback. Together, parts 1A and 1B have produced a data-driven, participant-informed intervention for evaluation in part 2, a successor pilot RCT that is currently in development. The findings from these pilot trials are expected to support a larger, multisite RCT of the intervention compared to usual care.

### Comparison With Prior Work

The RESET in ALL trial addresses 2 critical research gaps in behavioral interventions for eAYAs with cancer: lack of a specific focus on SB and beginning behavior change during therapy. The RESET in the ALL trial employs a 2-part design to address these research gaps. We chose to focus on SB because it comprises a large proportion of the day, is highly prevalent among eAYAs with cancer early in treatment, and often persists into survivorship, contributing to cardiometabolic complications, chronic disease, and premature mortality [2,3,15,22]. By intervening during therapy and integrating intervention activities during regularly scheduled clinic appointments, our approach supports a scalable, clinically integrated program that may improve health outcomes.

In ALL, the most common malignancy in this age group, excessive SB may exacerbate treatment-related toxicities and lead to poorer outcomes [6,18-20]. Additionally, prior intervention studies in pediatric oncology have largely targeted MVPA, an independent health behavior only weakly correlated with SB [13,14], and PA-focused studies in CCS have not consistently reduced SB [37,65]. In contrast, evidence from healthy youth suggests that interrupting sitting or reallocating even 30 minutes/day of SB to light activity can improve metabolic and psychosocial outcomes [23,25-27], supporting SB as a more feasible and potentially impactful intervention target during ALL therapy. The maintenance phase, during which acute toxicities typically subside, offers an ideal window for introducing such strategies.

To our knowledge, RESET in ALL is the first on-therapy, SB-focused intervention for eAYAs with ALL. Thus, there are no direct comparisons of our expected findings with prior trials. However, the two-part design, including an innovative app-based peer support group, offers a novel, stepwise approach to developing and optimizing mHealth interventions in pediatric and AYA oncology. Given the high survival rates for eAYAs, intervening early with scalable, clinically integrated interventions is an attractive strategy for improving health outcomes in the decades of anticipated life after cancer.

### Strengths and Limitations

The protocol has several strengths, including the novel focus on SB, intervening during therapy as part of routine care to minimize participant burden, the inclusion of an app-based peer support group, and a diverse study population. There are also limitations that should be noted. Due to the pilot nature of this trial, we had a small sample size, which precludes drawing definitive conclusions about the efficacy of the intervention. There was also no control group to compare intervention effects against, and we only recruited from a single site.

### Future Directions

Findings from part 1A of the trial were used to optimize part 1B of the trial. Both part 1A and part 1B will inform part 2 of the trial. If the subsequent pilot trials show promising results, a fully powered, multisite RCT will be conducted to confirm the efficacy of RESET to reduce SB across diverse eAYA clinical populations undergoing treatment. This definitive trial will compare RESET to usual care and will incorporate a longer follow-up period to understand the sustainability of effects.

### Conclusions

This protocol describes the first multicomponent mHealth intervention to reduce SB metrics in eAYAs undergoing cancer treatment. Using a sequential design, whereby prior trials inform the optimization of subsequent trials, we aim to develop and refine a scalable, clinically integrated approach to behavior change during treatment that enhances health outcomes in eAYA populations.

## Supplementary material

10.2196/107278 Multimedia Appendix 1 Details of protocol amendments and registry updates.

10.2196/107278 Checklist 1 SPIRIT 2022 checklist.

10.2196/107278 Peer Review Report 1 Funder Peer Review of RESET in ALL Study.

## Abbreviations

ALL: acute lymphoblastic leukemia
B-LLy: B-cell lymphoblastic lymphoma
CCS: childhood cancer survivors
CHLA: Children’s Hospital Los Angeles
COG: Children’s Oncology Group
CONSORT: Consolidated Standards of Reporting Trials
eAYA: adolescent and early young adult
EMR: electronic medical record
HAPA: Health Action Process Approach
HbA_1c_: hemoglobin A_1c_
HRQoL: health-related quality of life
hs-CRP: high sensitivity C-reactive protein
IPAQ: International Physical Activity Questionnaire
MET: metabolic equivalent of task
mHealth: mobile health
MPAL: mixed phenotype acute leukemia
MVPA: moderate to vigorous physical activity
PA: physical activity
PACE: Patient-Centered Assessment and Counseling for Exercise
PedsQL: Pediatric Quality of Life Inventory
QoL: quality of life
RCT: randomized controlled trial
SB: sedentary behavior
SPIRIT: Standard Protocol Items: Recommendations for Interventional Trials
T-LLy: T-cell lymphoblastic lymphoma
WtHR: waist-to-height ratio
