# Supplementary material for: A Mobile Health Intervention to Reduce Sedentary Behavior Among Adolescents and Early Young Adults Receiving Treatment for Acute Lymphoblastic Leukemia: Protocol for a Single-Arm Feasibility Trial
Source: JMIR Res Protoc. 2026 Oct 28;15:e107278. doi: 10.2196/107278 (PMC13717343; doi:10.2196/107278)
Supplement: Supplementary file 2 [file resprot-v15-e107278-s003.pdf]

St. Baldrick's FOUNDATION

Conquer Kids' Cancer

Applicant: Ivory, Brittany
Title: A Two-Part Clinical Trial to Implement and Optimize a Multi-Component Mobile Health Intervention to Reduce Sedentary Time in Adolescents with Acute Lymphoblastic Leukemia during Maintenance Therapy
Program: St. Baldrick's Foundation Fellowship
Institution: Children's Hospital Los Angeles
App #: 1357590

View Review Information

Close Window Print

Committee: Fellowships January 2025

Reviewer Role: Primary

Significance

Please score the significance the proposed research project. Does the candidate describe how the concepts, methods, technologies, treatments, services, or preventative interventions that drive this field will be changed if the proposed aims are achieved.:

The application comes from Dr. Brittany Van Remortel, a 3rd year fellow at CHLA who is proposing "A Two-Part Clinical Trial to Implement and Optimize a Multi-Component Mobile Health Intervention to Reduce Sedentary Time in Adolescents with Acute Lymphoblastic Leukemia during Maintenance Therapy." The significance of the proposal is high as we need innovative strategies to promote healthy behaviors among our growing population of survivors, particularly among those with ALL who have a high risk of obesity.

Candidate

Please score the candidate's background and preparation to undertake this project. Do they have appropriate experience and training? Is the applicant committed to a pediatric cancer research career? Does the candidate clearly describe their current career goals and the incremental education or skills to be attained during the grant period? Describe the

candidate's research efforts to this point in their research career, including any publications, prior research interests and experience.:
The applicant has a strong background and track record for the proposed work including prior work in physical activity interventions among adult cancer survivors and a community-based participatory research project on nutrition. Both efforts resulted in publications for the applicant including a primary author publication in Pediatrics. She was selected for the NCI-sponsored Transdisciplinary Research on Energetics and Cancer (TREC) Training Workshop. She has strong letters of recommendation.

Sponsor/Mentor
Please describe the qualifications of the sponsor/mentor. Do they have previous experience as a research supervisor? Do they have the ability to guide the candidate? Did they provide a plan that describes the nature of the supervision and mentoring that will occur during the proposed award period? Did they provide a plan for career progression for the candidate to move from the mentored stage of their career to independent research investigator status during the project period of the award?:
She has a complementary team of mentors including Dr. David Freyer (survivorship/AYA) and Dr. Britni Belcher (sedentary behavior). Dr. Freyer provides expertise on the population, design and implementation of the project while Dr. Belcher provides topical expertise on physical activity and sedentary behavior interventions.

Research Plan
Please score the quality and scientific rigor along with importance of the proposed research. Are the proposed research questions, design, and methodology of significant scientific and technical merit? Did the candidate address potential weaknesses? As applicable, did the candidate present plans to ensure a robust and unbiased approach? As applicable, did the candidate present plans to address relevant biologic variables, such as sex, for studies in vertebrate animals or human subjects? Is the research plan appropriate to the candidate's stage of research development and as a vehicle for developing the research skills described in the career development plan?:
The applicant describes the technique and endpoints for measuring feasibility, acceptability, and effects of the intervention. One potential pitfall is accrual, and the applicant describes appropriate alternative approaches. The applicant does not explore how physical comorbidities may impact the proposed intervention and how mental health (anxiety, depression, post-traumatic stress) could moderate the impact of the intervention.

Innovation
Please score the innovation of the project. Does the application challenge or seek to shift current research or clinical practice paradigms?:
Innovations of the application include the timing of the intervention during maintenance therapy and the target of reducing sedentary behaviors – a strategy which has been associated with improved metabolic function in other childhood populations (non-cancer) published by her co-mentor. Both are relevant questions for our field and could inform practice.

Environment and Resources
Please score the institutional resources and facilities available for this research. Does the institution provide a strong, well-

established pediatric cancer research and career development program? Is the institutional research environment well suited for the candidate's research plan? Is there institutional commitment to the candidate?:
The environment has a history of successful cancer research, and the team is well suited for the proposed research. The proposed work has been supported by a seed grant and has already accrued 1 patient.

Resource Sharing Plan
Please comment on the reasonableness of the resource sharing plan. Or is the rationale for not sharing resources reasonable? If you have additional suggestions related, please include them. No score required:
The applicant will follow the NIH Data Management and Sharing Policy for sharing the datasets generated from this work including sharing de-identified data.

Budget
Is the budget reasonable? No score required.:
The budget is reasonable.

Reviewer Role: Secondary

Significance
Please score the significance the proposed research project. Does the candidate describe how the concepts, methods, technologies, treatments, services, or preventative interventions that drive this field will be changed if the proposed aims are achieved.:
Interventions to reduce sedentary behaviors are important to combat the risks of obesity, impaired glucose metabolism, and cardiovascular disease in childhood cancer survivors, important risk factors for relapse and chronic disease and morbidity among survivors.

Candidate
Please score the candidate's background and preparation to undertake this project. Do they have appropriate experience and training? Is the applicant committed to a pediatric cancer research career? Does the candidate clearly describe their current career goals and the incremental education or skills to be attained during the grant period? Describe the candidate's research efforts to this point in their research career, including any publications, prior research interests and experience.:
The candidate is a 3rd-year fellow at CHLA, following and MD and MPH at the Univ of Wisconsin, and residency at CHOP. She has an impressive research track record for her level of training. During her MPH research, she was on a team that conducted a pilot trial testing feasibility and effect of a multi-level, technology-based

intervention to increase physical activity in breast and colorectal cancer survivors. As a resident, she co-led research to evaluate facilitators and barriers to produce consumption and WIC benefit redemption among low-income communities in

Philadelphia, and then developed a summer produce delivery program and conducted a randomized trial testing the effect of price on participation and perceived produce access. Both projects led to first-author publications. She was also selected for a competitive NCI training workshop on transdisciplinary research on energetics and cancer.

Sponsor/Mentor

Please describe the qualifications of the sponsor/mentor. Do they have previous experience as a research supervisor? Do they have the ability to guide the candidate? Did they provide a plan that describes the nature of the supervision and mentoring that will occur during the proposed award period? Did they provide a plan for career progression for the candidate to move from the mentored stage of their career to independent research investigator status during the project period of the award?:

The mentors are outstanding, with clear success in their field of research and as mentors, and their expertise is highly complementary. Dr David Freyer is a leader in the areas of cancer survivorship and AYA oncology, with extensive past and current NIH and other funding. He is Professor of Clinical Pediatrics, Medicine, and Population and Public Health Sciences at the Keck School of Medicine of USC; Director of the Survivorship & Supportive Care Program, Co-Director of the Adolescent and Young Adult (AYA) Cancer Program; and past Chair of the COG AYA Oncology Discipline Committee, and member of the Outcomes & Survivorship and Cancer Control & Supportive Care Steering Committees. He is also a recipient of an institutional faculty mentoring award.

Dr Britni Belcher is an Associate Professor in the Keck School of Medicine Department of Population and Public Health Sciences, with a research focus on physical

activity and sedentary behavior measurement, and their relationships with chronic disease risk factors. She has an extensive funding and publication record and an outstanding mentorship record.

Both mentors have written glowing letters of recommendation. Dr Freyer affirms his "strongest recommendation" and Dr Belcher states that the candidate is among the top trainees in her nearly 10 years of experience. They have been working closely with the candidate already, and present a detailed mentorship plan.

Research Plan

Please score the quality and scientific rigor along with importance of the proposed research. Are the proposed research questions, design, and methodology of significant scientific and technical merit? Did the candidate address potential weaknesses? As applicable, did the candidate present plans to ensure a robust and unbiased approach? As applicable, did the candidate present plans to address relevant biologic variables, such as sex, for studies in vertebrate animals or human subjects? Is the research plan appropriate to the candidate's stage of research development and as a vehicle for developing the research skills described in the career development plan?:

This is an outstanding study proposing to investigate an intervention on sedentary behaviors (SBs) in patients 12-18 years old receiving maintenance therapy for ALL. The 10-week, multicomponent mHealth intervention, called ReSeT, will use a Fitbit with inactivity-triggered prompts to move, individualized health coaching, and an app-based peer support group during a single cycle of maintenance therapy. Part 1 of the study will determine feasibility and acceptability in 30 non-randomized participants. Using these data and participant feedback, ReSeT will be optimized and then evaluated in Part 2 of the study via a 1:1 randomized pilot study of 20 participants (ReSeT vs usual care) to validate acceptability and evaluate trends of efficacy.

The proposal is clearly written and compelling. The study design is strong, with ambitious but attainable goals, and will form a solid foundation for a future larger, definitive trial. Conducting this trial will clearly strengthen the candidate's skills for future work in this area.

Innovation

Please score the innovation of the project. Does the application challenge or seek to shift current research or clinical practice paradigms?:

The study is distinctive in its focus on SBs rather than physical inactivity. Reducing prolonged SBs has distinct health benefits independent of encouraging periods of physical activity. It is also innovative in intervening during ALL maintenance therapy rather than waiting until patients are off therapy; and will leverage a novel app-based peer support group (GroupMe).

Environment and Resources

Please score the institutional resources and facilities available for this research. Does the institution provide a strong, well-established pediatric cancer research and career development program? Is the institutional research environment well suited for the candidate's research plan? Is there institutional commitment to the candidate?:

The resources at USC and CHLA are outstanding, including a well-developed research infrastructure, and a large patient population to power the study.

Resource Sharing Plan

Please comment on the reasonableness of the resource sharing plan. Or is the rationale for not sharing resources reasonable? If you have additional suggestions related, please include them. No score required:

no concerns

Budget

Is the budget reasonable? No score required.:

no concerns

Reviewer Role: Tertiary

Significance

Please score the significance the proposed research project. Does the candidate describe how the concepts, methods, technologies, treatments, services, or preventative interventions that drive this field will be changed if the proposed aims

are achieved.:
The candidate provides a clear description of how the interventions evaluated in this proposal could lead to significant practice changes.

Candidate
Please score the candidate's background and preparation to undertake this project. Do they have appropriate experience and training? Is the applicant committed to a pediatric cancer research career? Does the candidate clearly describe their current career goals and the incremental education or skills to be attained during the grant period? Describe the candidate's research efforts to this point in their research career, including any publications, prior research interests and experience.:
Dr. Van Remortel is an outstanding applicant. She demonstrates clear and focused commitment to her career development as a clinician-scientist focused on developing practice-changing supportive care interventions that improve short and long-term health among children and adolescents with cancer.

Sponsor/Mentor
Please describe the qualifications of the sponsor/mentor. Do they have previous experience as a research supervisor? Do they have the ability to guide the candidate? Did they provide a plan that describes the nature of the supervision and mentoring that will occur during the proposed award period? Did they provide a plan for career progression for the candidate to move from the mentored stage of their career to independent research investigator status during the project period of the award?:
Drs. Freyer and Belcher are outstanding mentors for this application. As stated by the applicant, they constitute an ideal team and provide complementary expertise to ensure the success of the proposed project. Dr. Freyer has an outstanding record of successfully training mentees. Belcher also demonstrates an excellent record of successful mentorship.

Research Plan
Please score the quality and scientific rigor along with importance of the proposed research. Are the proposed research questions, design, and methodology of significant scientific and technical merit? Did the candidate address potential weaknesses? As applicable, did the candidate present plans to ensure a robust and unbiased approach? As applicable, did the candidate present plans to address relevant biologic variables, such as sex, for studies in vertebrate animals or human subjects? Is the research plan appropriate to the candidate's stage of research development and as a vehicle for developing the research skills described in the career development plan?:
In this application, Dr. Van Remortel describes her multicomponent heath intervention, ReSeT to help change sedentary behaviors in patients 12-18y receiving maintenance therapy for ALL. Part 1 of the study will determine feasibility and acceptability in 30 non-randomized participants. Using these data and participant feedback, ReSeT will be optimized and then evaluated in Part 2 of the study via a 1:1 randomized pilot study of 20 participants (ReSeT vs usual care) to validate acceptability and evaluate trends of efficacy. The study design is based on known and successful principles of behavior change. The applicant presents a well-written and clear description of the project that includes an thoughtful approach to anticipated problems/pitfalls.

Of note, Part 1 of the study is currently ongoing with first patient enrolled August 2024. The applicant has received funding towards this project between 6/1/24 and 6/30/25. The applicant reported no overlap, this is likely due to the time period of the grant award. Given the numbers proposed and the patient population, this reviewer has no concerns regarding the funding for part 1 from 6/24-6/25

Innovation

Please score the innovation of the project. Does the application challenge or seek to shift current research or clinical practice paradigms?:

The lessons learned from this project or the successful implementation of the intervention has the potential to change clinical practice paradigms. Interventions to change sedentary behaviors and increase physical activity DURING treatment are critical to reduce short term and long term toxicities of current therapies. While the study population is appropriately homogeneous (maintenance therapy for ALL), if successful the intervention could be used in children/adolescents/young adults with various malignancies.

Environment and Resources

Please score the institutional resources and facilities available for this research. Does the institution provide a strong, well-established pediatric cancer research and career development program? Is the institutional research environment well suited for the candidate's research plan? Is there institutional commitment to the candidate?:

The environment and resources described are outstanding for this project.

Resource Sharing Plan

Please comment on the reasonableness of the resource sharing plan. Or is the rationale for not sharing resources reasonable? If you have additional suggestions related, please include them. No score required:

The resource sharing plan is appropriate.

Budget

Is the budget reasonable? No score required.:

The budget is reasonable.
