# Supplementary material for: A Mobile Health Intervention to Reduce Sedentary Behavior Among Adolescents and Early Young Adults Receiving Treatment for Acute Lymphoblastic Leukemia: Protocol for a Single-Arm Feasibility Trial
Source: JMIR Res Protoc. 2026 Oct 28;15:e107278. doi: 10.2196/107278 (PMC13717343; doi:10.2196/107278)
Supplement: Supplementary file 3 [file resprot-v15-e107278-s001.docx]

| **Supplemental Table S1**. Details of protocol amendments and registry updates. | | | | | |
| --- | --- | --- | --- | --- | --- |
| **Protocol Version Number** | **IRB^a^ Date** | **NCT^b^ Date** | **Reason for Changes** | **Number Enrolled** | **Change Occurred Before Outcome Review** |
| 1 | 12/16/2023 | 12/13/2023 | IRB: Initial submission.  ClinicalTrials.gov: Initial submission. | 0 | Yes |
| 2 | 4/4/2024 | None | Changes requested by division reviewers. We removed the randomization scheme to reflect the transition to a single-arm study. | 0 | Yes |
| 3 | 4/17/2024 | None | IRB staff preliminary review required changes to the initial application as follows: we updated funding descriptions, further described the fitness tracker (Fitbit), clarified how we would de-identify participant surveys, clarified the participant recruitment pool, and described data retention and direct identifiers. | 0 | Yes |
| 4 | 4/25/2024 | None | Changes requested by the IRB staff. We updated the fitness tracker and accelerometer as investigational devices with non-significant risk. We clarified there could be a small risk of injury from physical activity, data and specimen banking, and clarified confidentiality measures for exit interviews. | 0 | Yes |
| 5 | 5/10/2024 | None | Protocol approved with contingencies. We clarified the expected number of hours participants would spend on study activities, clarified procedures around using the app platform for peer support group, and updated the privacy information for exit interviews. | 0 | Yes |
| 6 | 5/23/2024 | None | IRB approval with contingencies. The protocol was edited to state that participants would need to create a new WhatsApp account to participate in the peer support group in order to protect privacy. If that is not possible, we would create a deidentified phone number using TextNow so the participants could create a new anonymous WhatsApp account.  This was the first protocol approved by the IRB. | 0 | Yes |
| 7 | 06/20/2024 | 7/16/2024 | IRB: Eligibility Revisions & Incentives. Prior to enrolling any participants, we revised the eligibility criteria. The study inclusion criteria were modified to more clearly define which leukemia diagnoses are eligible for the study and add clarification on timing of eligibility for patients treated with nelarabine. Diagnoses of diabetes mellitus and autoimmune/rheumatologic disorders were removed as exclusion criteria, given the presence of these diagnoses would not interfere with examination of the primary and secondary study aims; analysis of lab values can be adjusted based on patient's underlying medical conditions to accurately assess for trends during the intervention period. Patients with diabetes stand to benefit from participation in the intervention and were felt to be an important group to include in the study. The informed consent/assent and simplified assent forms were revised to add that participants will be paid with a $50 gift card for participating in the study.  ClinicalTrials.gov: Study information was changed to reflect single arm study, eligibility criteria was updated, study duration was extended, and the study status was set to ‘Recruiting’. | 0 | Yes |
| 8 | 8/07/2024 |  | IRB: App Change for Peer Support Group. We changed the app platform for the peer support group to provide stronger privacy for participants. This was routed to IT Security and then approved by IRB. | 2 | Yes |
| 9 | 11/19/2024 | 12/20/2024 | IRB: Age Limit Increase, Phone Consent, & Parent Survey. We increased the study participant upper age limit from 18 to 21 years to increase the number of patients eligible to enroll in the study and added an age-appropriate PedsQL survey, edited consent to allow for remote consenting over the phone or Microsoft Teams, and added a parent survey on study experiences to help inform trial optimization.  ClinicalTrials.gov: Study information was changed to reflect age limit increase. | 8 | Yes |
| 10 | 3/05/2025 |  | IRB: Eligibility Expansion & Enrollment Change. We edited the total number enrolled from 30 to 24 to account for 20% attrition and attain 20 evaluable participants within the trial timeline. Inclusion criteria were modified to allow patients to enroll after they have completed 1 month of maintenance chemotherapy rather than 1 cycle (3 months total), and patients receiving nelarabine as part of their maintenance chemotherapy regimen (T-ALL/T-LLy) are eligible to enroll while still completing nelarabine-containing cycles. This change is being made to allow patients to enroll on study earlier in maintenance chemotherapy given patients are often doing well at this time and acute toxicities have often improved, including while receiving nelarabine. | 15 | Yes |
| 11 | 7/17/2025 | 10/10/2025  1/20/2026  7/1/2026 | IRB: PI Name and Contact Info Change, and Funding Change. The study PI legally changed her last name. This information was updated for all contact information and consent/assent forms. Funding was updated to reflect that as of July 1, 2025, the study is being funded by a Clinical Research Fellow Grant awarded to the PI from the Rally Foundation for Childhood Cancer Research (non-profit foundation). Funding from the Nautica Fund ended as of June 30, 2025.  NCT: Study status was changed to ‘Active, not recruiting’, the estimated study end date was extended, funding was updated, and PI title was updated.  NCT: Study status was changed to ‘Completed’, the estimated study end date was changed to actual end date.  NCT: Study primary completion date was updated. | 18 | Yes |
| Table describing edits and amendments to each protocol version and ClinicalTrials.gov updates.  ^a^IRB: Institutional Review Board.  ^b^NCT: National Clinical Trial. | | | | | |
